# Large variations in ocular dimensions in a multiethnic population with similar genetic background

**DOI:** 10.1038/srep22931

**Published:** 2016-03-07

**Authors:** Zhiqiang Niu, Jun Li, Hua Zhong, Zhonghua Yuan, Hua Zhou, Yang Zhang, Yuansheng Yuan, Qin Chen, Chen-Wei Pan

**Affiliations:** 1 Department of Ophthalmology, the First Affiliated Hospital of Kunming Medical University, Kunming, China; 2 Department of Ophthalmology, the Second People’s Hospital of Yunnan Province, Kunming, China; 3 Department of Ophthalmology, the First People’s Hospital of Kunming City, Kunming, China; 4 Department of Ophthalmology, the First Affiliated Hospital of Nanjing Medical University, Nanjing, China; 5 Jiangsu Key Laboratory of Preventive and Translational Medicine for Geriatric Diseases, School of Public Health, Medical College of Soochow University, Suzhou, China

## Abstract

We aimed to describe the ethnic variations in ocular dimensions among three ethnic groups with similar genetic ancestry from mainland of China. We included 2119 ethnic Bai, 2202 ethnic Yi and 2183 ethnic Han adults aged 50 years or older in the study. Ocular dimensions including axial length (AL), anterior chamber depth (ACD), vitreous chamber depth (VCD) and lens thickness (LT) were measured using A-scan ultrasonography. Bai Chinese had longer ALs (P < 0.001), deeper ACDs (P < 0.001) but shallower VCDs (P < 0.001) compared with the other two ethnic groups. There were no ethnic variations in LTs. Diabetes was associated with shallower ACDs and this association was stronger in Bai Chinese compared with Yi or Han Chinese (P for interaction = 0.02). Thicker lenses were associated with younger age (*P* = 0.04), male gender (*P* < 0.001), smoking history (*P* = 0.01), alcohol intake (*P* = 0.03), the presence of cataract (*P* < 0.001), and the presence of diabetes (*P* < 0.001). There were significant differences in ocular dimensions among different ethnic groups with small differences in genetics but large variations in cultures and lifestyles.

Numerous studies have tried to elucidate the etiologies of age-related eye disorders including refractive errors, age-related macular degeneration, age-related cataract and glaucoma, which are the major causes of visual impairment and blindness in older people^1^. Potential ethnic variations in the risk of these age-related eye disorders have been reported. For example, myopia seems to be more prevalent in East Asians including Chinese, Japanese or Koreans^2^,^3^. The prevalence of primary open angle glaucoma is higher in Africans while the prevalence of primary angle closure glaucoma is higher in Asians compared with Europeans^4^. Anatomically, these age-related eye disorders have been linked with ocular dimensions such as axial length (AL)^5^. Therefore, an in-depth knowledge of the potential ethic variations in ocular dimensions provides further insights into the etiologies of these eye disorders.

Up till now, population-based studies have reported a wide range of normative data on ocular dimensions among various ethnic groups in different areas of the world^6^,^7^,^8^,^9^,^10^,^11^. However, inter-study comparisons of these estimates are difficult considering the methodological disparities among different studies including sampling strategies, age ranges of the study subjects, and measurements of ocular dimensions. The Singapore Epidemiology of Eye Diseases (SEED) study provided initial evidence on a possible ethnic difference in the ocular dimensions measured by IOL master among the three major ethnic groups including Chinese, Malays and Indians in this city state by collecting data using the same study protocols^12^. It is interesting to understand whether ethnic differences exist in multiethnic population with relatively smaller variations in genetics. In addition, IOL master does not provide important ocular dimension data of lens thickness (LT) or vitreous chamber depth (VCD) and whether there are ethnic differences in these ocular dimensions remains unclear.

China is the world’s most populous country with a multiethnic population including Han ethnicity and another 55 ethnic minorities. Han ethnicity is the major ethnic group which accounts for about 90% of the entire national population. The Yi and Bai ethnicities are the major ethnic minorities in China. These ethnic groups have different cultures, languages and lifestyles, yet relatively similar genetic ancestry^13^. In this study, we describe the ethnic variations in the distributions and determinants of ocular dimensions measured by an Echoscan among the three ethnic groups (Bai, Yi and Han ethnicities) in rural China.

## Methods

### Study cohort

The Yunnan Minority Eye Studies (YMES) are population-based studies conducted among different ethnic groups including the Han ethnicity (the major ethnic group) and other ethnic minorities in Southwestern China using the same study protocols for data collection. The YMES were conducted from 2011 to 2014. In a previous report, we have described the detailed methodology of YMES and some major findings in a single ethnic group, that is, the Bai ethnicity^14^,^15^,^16^,^17^. Now, we have finished the data collection for the other two ethnic groups: Yi and Han ethnicities, which allowed for multiethnic comparisons. Briefly, random cluster sampling strategies were adopted to select ethnic Bai, Yi and Han adults aged 50 years or older living in a rural community. These three ethnic groups resided in separate towns within a rural community in Yunnan Province. Information regarding ethnicity was collected from the study participants’ identity cards. Different ethnic groups were not living in the same villages and there were few inter-marriages among different ethnic groups. Each village in the study site with a population of approximately 1000 was considered as a cluster during the sampling procedure. Villages with a population of less than 750 were combined and those of more than 1500 were divided and regrouped. Subsequently, 10% of the total clusters were randomly selected using a computer-assisted program. In the end of the study, 2133 (77.8%) ethnic Bai, 2208 (82.0%) ethnic Yi and 2205 (80.5%) ethnic Han adults participated in this study, respectively. There were no age or gender differences between study participants and non-participants among all ethnic groups (all P > 0.05). The same research team collected data from all three ethnic groups using the same equipment.

All studies were approved by the Kunming Medical University Institutional Review Board and the conduct of the studies adhered to the Declaration of Helsinki. The study methods were carried out in accordance with the approved guidelines. Informed consent was obtained from all study participants.

### Clinical Examinations

Ocular dimensions including AL, anterior chamber depth (ACD), VCD and LT were measured using an Echoscan (US-800; Nidek Co., Ltd, Tokyo, Japan) and the mean of the 5 readings was used for data analysis. Non-cycloplegic autorefraction was performed using an autorefractor (RM-8000; Topcon Corp., Tokyo, Japan) and refraction was subjectively refined until the best visual acuity was obtained.

Information regarding participants’ educational level, socioeconomic status, lifestyle-related factors (e.g., smoking, alcohol intake), disease history, and medication intake was collected using a detailed questionnaire by a trained research assistant. Height was measured in centimeters using a wall-mounted measuring tape after removing shoes while weight was measured in kilograms after taking off heavy clothing. Systolic blood pressure, diastolic blood pressure and pulse rate for all participants were recorded using a standardized mercuric-column sphygmomanometer, and one of four cuff sizes (pediatric, regular adult, large, or thigh) was selected based on the circumference of the participant’s arm. Hypertension was defined as systolic blood pressure ≥140 mmHg, diastolic blood pressure ≥90 mmHg, or a physician diagnosis. Diabetes mellitus was defined as non-fasting glucose levels higher than 200 mg/dL (11.1 mmol/L) or previous physician diagnosis of diabetes or use of anti-diabetic medications based on the American Diabetes Association guidelines^18^. Slit-lamp examination (model SL-1E; Topcon) was performed by trained study ophthalmologists and included a clinical grading of cataract according to the Lens Opacities Classification System (LOCS) III^19^.

### Statistical analyses

Data analyses were performed using STATA version 11.0 (StataCorp, College Station, Tex., USA). Since ocular dimensions for both eyes were highly correlated (Pearson correlation coefficient for AL = 0.96, *P* < 0.001; ACD = 0.91, P < 0.001; VCD = 0.95, P < 0.001; LT = 0.90, P < 0.001) and the results of analysis for both eyes were similar, results were presented for right eyes only.

Mean ocular dimension parameters were compared across each age group using the Analysis of Variance (ANOVA) test. Age-sex-adjusted mean values were calculated using a covariance model. Associations between factors of interest and ocular dimension parameters were initially assessed using univariate regression analyses. Besides age, sex and ethnicity, factors with a P value of less than 0.10 in univariate models were retained in the multivariate models. To determine whether ethnicity modified associations between factors of interest and a specific ocular dimension parameter, a linear regression model was established with interaction terms between ethnicity and each potential risk factor, and a likelihood ratio test was performed on the interaction terms. If the interaction term was statistically significant (P < 0.05), stratified analysis were performed subsequently.

## Results

Table 1 compares the demographic and systematic parameters among the three ethnic groups. In brief, adults of Bai ethnicity were youngest, best educated, heaviest, had the highest blood pressure and were least likely to smoke or take alcohol drinks. Adults of Yi ethnicity were shortest, lightest, and were most likely take alcohol drinks. Adults of Han ethnicity had the lowest blood pressures.

**Figure 1:**
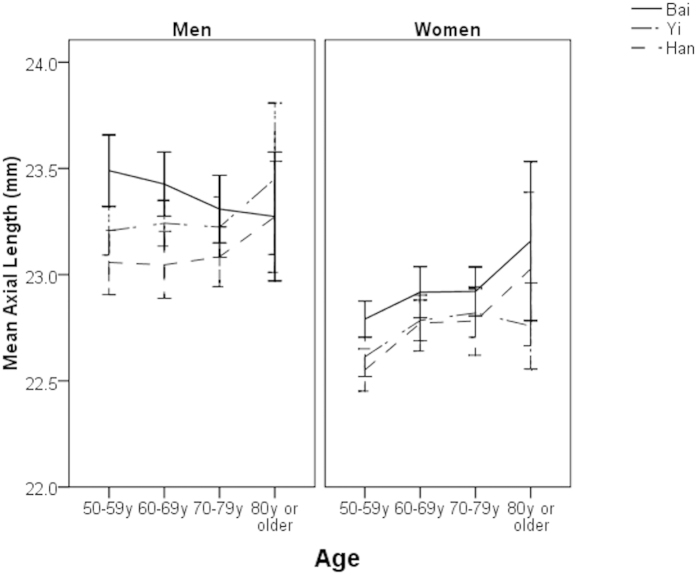
Mean axial length by age, sex and ethnicity.

**Figure 2:**
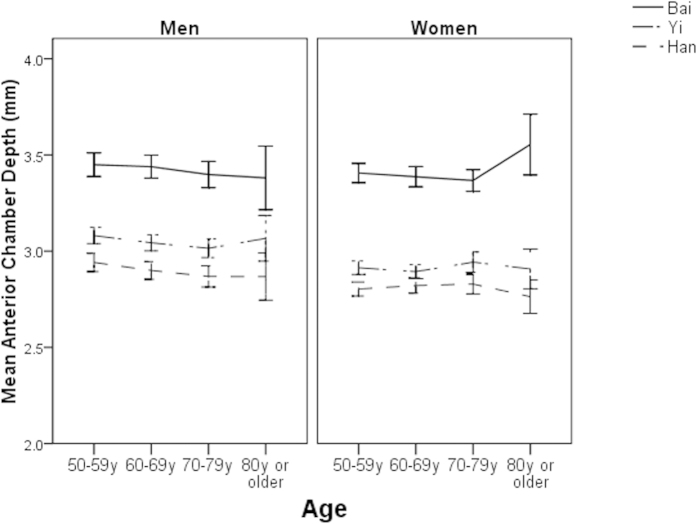
Mean anterior chamber depth by age, sex and ethnicity.

**Figure 3:**
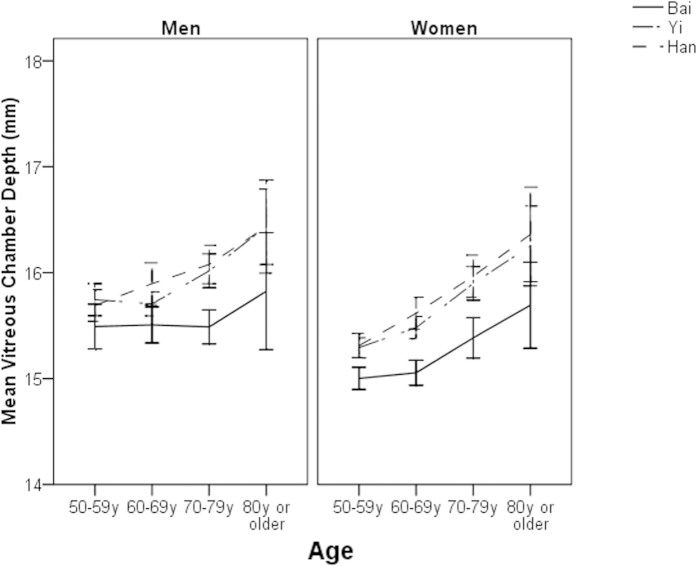
Mean vitreous chamber depth by age, sex and ethnicity.

**Figure 4:**
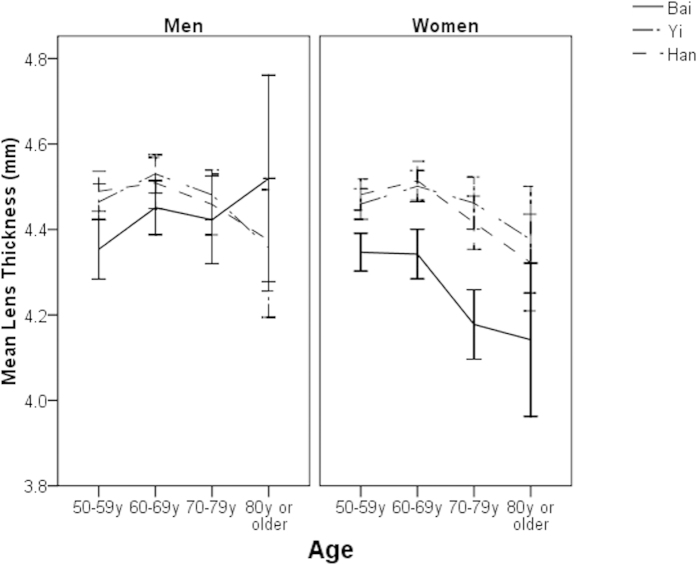
Mean lens thickness by age, sex and ethnicity.

Of the total 6546 study participants, 63 who had missing data in the right eye were excluded, leaving 6483 (99%) for further analysis. ALs, ACDs, and VCDs were not normally distributed in any ethnic groups (all P for K-S test < 0.05). LTs followed a normal distribution in all ethnic groups (all P for K-S test > 0.05). The mean age- and sex-adjusted AL were 23.15 ± 1.23 mm (95% confidence interval [CI] 23.11, 23.20), 23.04 ± 1.03 mm (95% CI 23.00, 23.08) and 22.95 ± 1.21 mm (95% CI 22.86, 22.95) in ethnic Bai, Yi and Han adults, respectively (P < 0.001). These figures were 3.42 ± 0.54 mm (95% CI 3.38, 3.46), 2.98 mm ± 0.38 (95% CI 2.94, 3.02) and 2.86 ± 0.40 mm (95% CI 2.81, 2.90) for ACD (P < 0.001), 15.35 ± 1.35 mm (95% CI 15.31, 15.40), 15.73 ± 1.02 mm (95% CI 15.69, 15.77) and 15.70 ± 1.26 mm (95% CI 15.66, 15.74) for VCD (P < 0.001), and 4.35 ± 0.62 mm (95% CI 4.30, 4.39), 4.48 mm ± 0.43 (95% CI 4.44, 4.52) and 4.47 mm ± 0.48 (95% CI 4.45, 4.52) for LT (P = 0.01). After adjusting for the effect of height, the magnitude of differences in the mean values of ocular dimensions did not change significantly (Table 2). The age-specific mean ocular biometric data are shown in Figs 1, 2, 3, 4.

In univariate regression analyses, longer ALs were associated with older age (*P* < 0.001), male sex (*P* < 0.001), Bai (*P* < 0.001) ethnicity, formal education (*P* = 0.005), taller stature (*P* < 0.001), greater weight (*P* < 0.001), the presence of hypertension (*P* = 0.01), and the absence of smoking history (*P* < 0.001). Deeper anterior chambers were associated with male sex (*P* < 0.001), Bai (*P* < 0.001) or Yi (*P* < 0.001) ethnicity, taller stature (*P* < 0.001), formal education (*P* = 0.01), and the absence of diabetes (*P* < 0.001). Deeper vitreous chambers were associated with older age (*P* < 0.001), male sex (*P* < 0.001), Han ethnicity (*P* < 0.001), formal education (*P* < 0.001), taller stature (*P* < 0.001), greater weight (*P* < 0.001), and the presence of hypertension (*P* < 0.001). Thicker lenses were associated with younger age (*P* < 0.001), male sex (*P* < 0.001), formal education (*P* < 0.001), taller stature (*P* < 0.001), greater weight (*P* < 0.001), and the presence of smoking history (*P* < 0.001), alcohol intake (*P* = 0.001), the presence of cataract (*P* < 0.001), and the presence of diabetes (*P* = 0.004).

In multivariate linear regression models adjusting for age, sex, ethnicity and factors with a P value of less than 0.10 in univariate models, longer ALs were associated with older age, male sex, Bai or Yi ethnicity, formal education, taller stature, greater weight, and the absence of smoking history. Deeper anterior chambers were associated with male sex, Bai or Yi ethnicity, and the absence of diabetes. Deeper vitreous chambers were associated with older age, male sex, Han ethnicity, formal education, taller stature, and greater weight. Thicker lenses were associated with younger age, male sex, the presence of smoking history, alcohol intake, the presence of cataract, and the presence of diabetes (Table 3).

A significant joint effect of diabetes with ethnicity on ACD (P for interaction = 0.02) was detected using a likelihood ratio test. Further ethnicity-stratified analysis indicated the association of diabetes with ACD (regression coefficient = −0.039 mm for Bai ethnicity; regression coefficient = −0.025 mm for Yi ethnicity; regression coefficient = −0.017 mm for Han ethnicity) was stronger in adults of Bai ethnicity compared with those of Yi or Han ethnicities in multivariate analyses.

## Discussion

In this multiethnic population of adults aged 50 years or older, we found significant differences in ocular dimensions such as ALs, ACDs and VCDs but not LTs among various ethnic groups with small genetic differences. Adults of Bai ethnicity had the deepest anterior but shallowest vitreous chambers among the three ethnic groups. Further well-designed cohort studies are warranted to assess whether the observed ethnic variations in ocular dimensions are related to different risks of age-related eye disorders in this population.

The take-home message of our study was that people of different ethnicities with small genetic differences may also have large differences in ocular dimensions. Previous multiethnic studies have shown a significant ethnic variation in ocular dimensions among different ethnic groups, which was considered to be attributed to the variations in genetics among different ethnic groups. Although previous data indicated that genetic differences in ethnic groups in Southwestern China are relatively smaller^13^, it needs to be recognized that statements about commonality are based on the overall genome, and a close global relationship does not preclude very specific differentiation between populations. Thus, based on the data in this study, we could not conclude that interplay and effects of cultures, lifestyles, or socioeconomic status in early life might affect the growth of eyeball, resulting in different ocular dimensions in adulthood.

We observed that ethnic Bai people had the longest ALs after adjusting for height among the three ethnic groups. AL is known to be composed of ACD, VCD and LT and the longest ALs observed in Bai people were mainly explained by that they had the deepest ACDs. The explanation for this interesting finding was unclear. It is likely that Bai people were inherently born with a deeper ACD and a shallower VCD compared with other ethnic groups in this study. Further studies are warranted to examine whether the differences in ocular biometry observed in this study had functional implications. For example, shallower ACDs are associated with a higher risk of angle closure glaucoma. Therefore, whether Bai people are more susceptible to angle closure glaucoma compared with the other two groups needs to be investigated.

Another interesting finding of this study was that patients with diabetes were more likely to have shallower anterior chambers and this association was modified by ethnicity. A recent meta-analysis of 47 individual studies indicated that diabetes is an independent risk factor for glaucoma^20^. Some individual studies have also reported that diabetes could increase the risk of open angle glaucoma^21^,^22^. Considering that the magnitude of difference was small, we cannot conclude that diabetes is a risk factor for angle closure glaucoma. Further well-designed cohort studies are warranted to confirm this finding and basic research is needed to elucidate the biological mechanism behind this association.

We observed a slightly increasing trend of AL and VCD with age in this study. VCD is a major part of AL. This age-related pattern of AL was different from other studies in Asians living in urban areas, which revealed an inverse relationship between older age and increasing AL^6^,^7^,^23^. Considering that AL is a major determinant for refractive status, the namely “cohort effect” on myopia prevalence found in many other population-based studies^12^,^24^,^25^,^26^,^27^,^28^ was not observed in this study. The possible explanation for this result is that the study area is an inland rural town located in the southwest part of China and has not experienced a dramatic change in social and environmental factors during the past few decades as compared with many metropolises in China and other countries in East Asia.

There were few studies exploring the ethnic variations in LTs and their associated factors. We found that ethnic variations in LTs were not significant after adjusting for the effects of a wide range of confounders. The major determinants for LTs are lifestyle-related factors (e.g. smoking, alcohol intake) or metabolic disorders such as cataract or diabetes, suggesting that metabolism plays a major role in crystalline lens growth. Smoking is a well-established risk factor for cataract^29^. Heavy alcohol drinking was found to be associated with cataract in a recently published meta-analysis^30^. There is a need to identify the specific metabolic biomarkers which are linked with the growth of crystalline lens. In addition, it is a surprising to find that LTs decreased slightly with increasing age, which contradicted previous view that LTs decrease with increasing age. However, the aged effect on LT was very small and the P value was marginally significant in this study (P = 0.04). This observation may be explained by the increases in height and biometry which overshadow the effects of longitudinal change. It was also likely to be a chance finding.

The study’s strengths included a large sample size, multiethnic study participants, reasonable response rates, standardized clinical measurements for ocular dimensions and the usage of the same study protocols for data collection across different ethnic groups. There were also some limitations for this study, which should be acknowledged. First, A-scan ultrasonography may not be precise in measuring some of the ocular dimensions such as LT, especially when cataract is present. Second, we were unable to identify the full set of explanatory factors for the observed ethnic differences in AL, ACD and VCD. Third, ethnicity was identified by official records in this study. Genetic measures of ancestry may be more accurate for ethnicity identification but was not performed in this study due to limited resources. Finally, the cross-sectional design limited the ability to assess causal relationship when analyzing risk factors.

In conclusion, this study of multiethnic participants living in the same geographic location in rural China found significant ethnic variations in ocular dimensions including ALs, ACDs and VCDs but not LTs, with Bai Chinese having deeper anterior but shallower vitreous chambers as compared with the other two ethnic groups. Our study also provided the normative data for ocular dimensions in ethnic minorities in China, which are important to guide future clinical practice (e.g., cataract or refractive surgery) or research (e.g., designing multi-ethnic clinical trials on AL or ACD-related conditions such as retinal disorders and glaucoma).

## Additional Information

**How to cite this article**: Niu, Z. *et al.* Large variations in ocular dimensions in a multiethnic population with similar genetic background. *Sci. Rep.* **6**, 22931; doi: 10.1038/srep22931 (2016).

## Figures and Tables

**Table 1:** Demographic and systemic parameters among the three ethnic groups.

	Bai ethnicity (n = 2119)	Yi ethnicity (n = 2202)	Han ethnicity (n = 2183)	P value
Demographic and systemic factors				
Age (years)	64.4 (9.7)	65.0 (9.2)	65.4 (9.4)	0.004
Female gender	1352 (63.8)	1254 (56.9)	1307 (59.9)	<0.001
No formal education	759 (35.8)	1140 (51.8)	1174 (53.8)	<0.001
Body mass index (kg/m^2^)	21.9 (5.3)	20.5 (5.9)	21.7 (7.9)	<0.001
Height (cm)	158.2 (11.5)	155.3 (8.5)	156.2 (8.2)	<0.001
Weight (kg)	54.8 (9.9)	49.6 (14.3)	52.9 (20.1)	<0.001
Systolic blood pressure (mmHg)	145.6 (25.6)	143.0 (26.0)	140.0 (24.8)	<0.001
Diastolic blood pressure (mmHg)	88.2 (17.8)	87.5 (19.1)	86.0 (17.2)	<0.001
Diabetes	48 (2.3)	52 (2.4)	57 (2.6)	0.07
Smoking history	539 (25.4)	694 (31.5)	702 (32.2)	<0.001
Alcohol intake	316 (14.9)	591 (26.8)	451 (20.7)	<0.001

Data presented are means (standard deviations) or number (%), as appropriate for variable.

**Table 2:** Ocular biometry among the three ethnic groups.

	Bai ethnicity	Yi ethnicity	Han ethnicity	P value
Axial length (mm)				
Age-sex-adjusted	23.15 (1.23)	23.04 (1.03)	22.95 (1.21)	<0.001
Age-sex-height-adjusted	23.16 (1.20)	23.10 (1.01)	22.98 (1.20)	0.001
Anterior chamber depth (mm)				
Age-sex-adjusted	3.42 (0.54)	2.98 (0.38)	2.86 (0.40)	<0.001
Age-sex-height-adjusted	3.35 (0.52)	3.01 (0.39)	2.90 (0.41)	<0.001
Vitreous chamber depth (mm)				
Age-sex-adjusted	15.35 (1.35)	15.73 (1.02)	15.70 (1.26)	0.008
Age-sex-height-adjusted	15.28 (1.32)	15.70 (1.01)	15.65 (1.29)	0.005
Lens thickness (mm)				
Age-sex-adjusted	4.35 (0.62)	4.48 (0.43)	4.47 (0.48)	<0.001
Age-sex-height-adjusted	4.39 (0.62)	4.48 (0.43)	4.47 (0.48)	<0.001

Data presented are means (standard deviations).

**Table 3:** Multivariate regression analyses on the determinants of ocular dimensions.

	Axial length (mm)			Anterior chamber depth (mm)			Vitreous chamber depth (mm)			Lens thickness (mm)		
	Beta	95% CI	P	Beta	95% CI	P	Beta	95% CI	P	Beta	95% CI	P
Age (per year increase)	0.009	0.006, 0.011	<0.001	−0.001	−0.004, 0.002	0.64	0.021	0.018, 0.024	<0.001	−0.003	−0.006, 0	0.04
Male vs. female	0.36	0.30, 0.43	<0.001	0.071	0.008, 0.134	0.03	0.20	0.14, 0.26	<0.001	0.079	0.015, 0.143	0.02
Ethnicity												
Bai	0.28	0.18, 0.38	<0.001	0.54	0.44, 0.64	<0.001	−0.29	−0.39, −0.19	<0.001	−0.09	−0.19, 0.01	0.08
Yi	0.12	−0.06, 0.18	0.20	0.12	0.06, 0.18	<0.001	0.05	−0.01, 0.11	0.12	0.001	−0.06, 0.062	0.97
Han		Ref			Ref			Ref			Ref	
No formal vs. formal education	−0.12	−0.18, −0.06	<0.001	−0.025	−0.123, 0.074	0.62	−0.10	−0.16, −0.04	<0.001	0.02	−0.04, 0.06	0.23
Height (per cm increase)	0.009	0.006, 0.011	<0.001	0.001	0.002, 0, 005	0.45	0.008	0.005, 0.011	<0.001	−0.002	−0.005, 0.001	0.24
Weight (per kg increase)	0.003	0.001, 0.005	<0.001		–		0.002	0, 0.004	0.014		–	
Hypertension (Yes vs. No)	0.08	−0.05, 0.13	0.34		–		0.06	−0.07, 0.11	0.58		–	
Diabetes (Yes vs. No)	0.12	−0.11, 0.34	0.55	−0.023	−0.028, −0.018	0.008		–		0.21	0.15, 0.27	<0.001
Cataract (Yes vs. No)		–			–			–		0.38	0.30, 0.46	<0.001
Smoking (Yes vs. No)	−0.16	−0.30, −0.02	0.002					–		0.35	0.27, 0.43	0.01
Alcohol drinking (Yes vs. No)		–			–			–		0.11	0.03, 0.19	0.03
